# Expression of Concern: The role of DOT1L in the proliferation and prognosis of gastric cancer

**DOI:** 10.1042/BSR-20193515_EOC

**Published:** 2021-04-06

**Keywords:** DOT1L, EPZ5676, Gastric cancer, Prognosis

The Editorial Office has been made aware of potential issues surrounding the scientific validity of this paper, hence has issued an expression of concern to notify readers whilst the Editorial Office investigates.

